# Association of neutrophil-percentage-to-albumin ratio with mortality in older stroke survivors

**DOI:** 10.3389/fnagi.2025.1611289

**Published:** 2025-05-30

**Authors:** Jie Huang, Xiaowei Zhang, Luyao Ding, Jiaxiang Yu, Mingshen Lin

**Affiliations:** ^1^Department of Cardiology, Lishui Municipal Central Hospital, Fifth Affiliated Hospital of Wenzhou Medical University, Lishui, China; ^2^Department of Clinical Laboratory, Lishui Municipal Central Hospital, Fifth Affiliated Hospital of Wenzhou Medical University, Lishui, China; ^3^Department of Emergency Medicine, Lishui Municipal Central Hospital, Fifth Affiliated Hospital of Wenzhou Medical University, Lishui, China

**Keywords:** NPAR, elderly, stroke survivors, mortality, NHANES

## Abstract

**Background:**

The neutrophil-percentage-to-albumin ratio (NPAR) functions as an integrative marker representing inflammatory response and nutritional health. However, its association with mortality in elderly stroke survivors has not been explored.

**Methods:**

This cohort study analyzed data from 1,026 elderly stroke survivors in the National Health and Nutrition Examination Survey (NHANES, 1999-2018). The association of NPAR with mortality was analyzed using Cox proportional hazards regression, restricted cubic splines (RCS), Kaplan-Meier survival analysis, and time-dependent receiver operating characteristic (ROC) curves. Subgroup analyses and interaction tests were also performed.

**Results:**

During the 6.65-year median follow-up, elevated NPAR showed independent associations with increased all-cause and cardiovascular mortality. Quartile-based analysis revealed 69 and 87% greater mortality hazards for the highest versus lowest NPAR groups, respectively. RCS analysis revealed a non-linear threshold effect at NPAR = 14.5, beyond which the risk of all-cause mortality increased sharply. NPAR demonstrated stable predictive accuracy, with time-dependent AUC ranging from 0.664 to 0.607 for all-cause mortality and 0.652-0.609 for cardiovascular mortality over 3-10 years. Subgroup analyses confirmed consistency across different sex, BMI, lifestyle habits, and comorbidity categories.

**Conclusion:**

This study underscores a strong positive correlation between NPAR and prognosis in older adult stroke survivors in the United States, indicating its potential as a novel biomarker for prognostic assessment.

## 1 Introduction

Stroke remains the second leading cause of mortality globally, with its burden continuing to escalate (Hilkens et al., 2024). Epidemiological models predict that stroke-related deaths will rise dramatically from 6.6 million cases in 2020 to 9.7 million by mid-century (Feigin et al., 2023). Concerningly, more than 50% of individuals who survive a stroke continue to suffer from lasting functional limitations while facing increased likelihood of subsequent cardiovascular complications (Hobeanu et al., 2022). The accelerating global demographic shift toward an aging population has led to marked increases in overall and cardiovascular-related mortality in older stroke survivors, creating significant burdens for medical services worldwide (Qu et al., 2024). Community-based studies in the United States report that the 5-year cumulative mortality rate among stroke survivors aged ≥ 65 years reaches 50%, with cardiovascular complications being the primary contributor (Bravata et al., 2007). Although traditional risk assessment tools are widely utilized in clinical settings, their predictive performance in elderly community-dwelling populations remains suboptimal (Coll et al., 2020). Moreover, these tools lack actionable biomarkers to guide personalized prevention and management strategies (Delgardo et al., 2023). Consequently, the early identification of potential risk factors for poor prognosis in older stroke survivors is crucial for developing effective prevention and intervention strategies.

The neutrophil-percentage-to-albumin ratio (NPAR) is a novel integrated inflammatory biomarker derived from routine blood tests, offering key advantages in accessibility and cost-effectiveness (Kurkiewicz et al., 2023). Its distinct value lies in its capacity to simultaneously capture two fundamental pathophysiological processes: fluctuations in neutrophil percentage, a critical component of innate immunity, serve as a sensitive indicator of systemic inflammation (Silvestre-Roig et al., 2019), while albumin levels not only reflect hepatic synthetic function but also provide a reliable surrogate for nutritional status (Spinella et al., 2016). Unlike traditional inflammatory markers, NPAR integrates inflammatory activation with metabolic-nutritional status, offering a more comprehensive framework for prognostic evaluation (Jiao et al., 2023). Emerging evidence has established strong associations between NPAR and adverse outcomes in multiple conditions, including heart failure (Wu et al., 2023), chronic obstructive pulmonary disease (Lan et al., 2023), diabetes (Jing et al., 2024), hypertension (Yang et al., 2025), metabolic dysfunction-associated fatty liver disease (Dong et al., 2024), and malignancies (Li et al., 2025). However, although NPAR represents a modifiable biomarker that may be influenced by interventions such as anti-inflammatory therapy and nutritional optimization, its prognostic significance in elderly stroke survivors remains largely unexplored.

Utilizing data from the community-based elderly stroke cohort within the National Health and Nutrition Examination Survey (NHANES), this study investigates the independent prognostic significance of NPAR for overall and cardiovascular mortality, providing insights into individualized risk assessment and integrated inflammation-nutrition intervention strategies.

## 2 Materials and methods

### 2.1 Data source and participants

Updated biennially, NHANES employs an innovative multimodal data collection framework comprising five key components: demographic characteristics, dietary intake, clinical examinations, laboratory analyses of biological specimens, and standardized questionnaire responses (Hu et al., 2020). A probability-based sampling design ensures nationally representative coverage across diverse geographic regions, racial and ethnic groups, and socioeconomic strata within the United States (Liu et al., 2022). The NCHS-IRB formally authorized this research, with mandatory written consent procedures completed for all enrollees during the pre-participation screening phase. As an open-access resource, NHANES provides a robust foundation for evidence-based public health policymaking and chronic disease prevention research.

**FIGURE 1:**
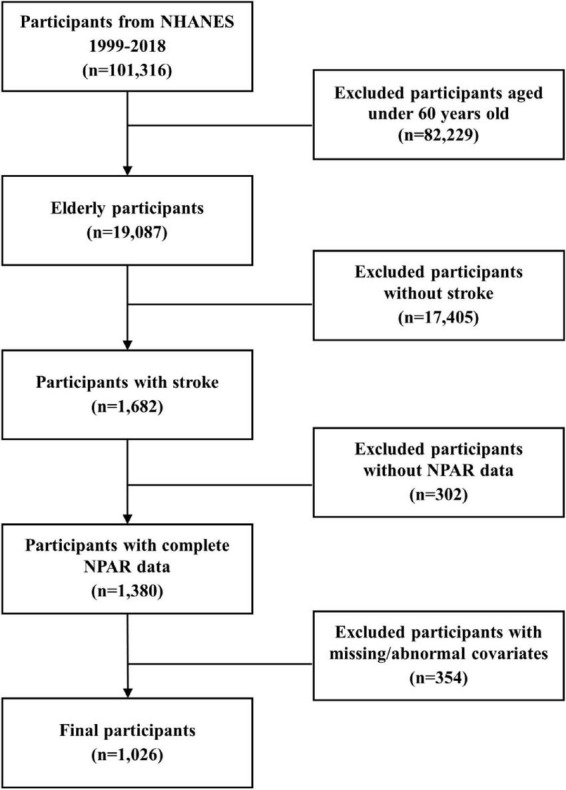
Flow chart of the study.

Utilizing data spanning 10 NHANES survey cycles (1999-2018), we identified 1,026 qualifying subjects after applying predetermined exclusion criteria: (1) age below 60 years; (2) no history of stroke; (3) incomplete survival data; (4) missing NPAR measurements; (5) incomplete covariate data or the presence of extreme outliers (Figure 1). Stroke history was determined using a standardized questionnaire item: “Were you ever diagnosed with a stroke by a physician or healthcare provider?” Affirmative responses classified individuals as stroke survivors. It is important to note that this assessment did not differentiate between stroke subtypes, such as ischemic or hemorrhagic stroke, and included asymptomatic cerebrovascular events identified through imaging. Previous studies have validated the accuracy and reliability of this self-reported method for capturing stroke history (Zhang et al., 2024).

### 2.2 Outcomes

Mortality status was ascertained by probabilistically linking NHANES participant records to the National Death Index (NDI) database. Follow-up commenced at the baseline assessment—defined as the date of the interview and physical examination—and continued until December 31, 2019. The primary outcome was all-cause mortality or censoring at the end of follow-up. Mortality causes were categorized according to ICD-10 criteria. Cardiovascular deaths encompassed those attributed to cardiac conditions (ICD-10: I00-I09, I11, I13, I20-I51) and cerebrovascular disorders (ICD-10: I60-I69) (Yu et al., 2024). All-cause mortality encompassed all confirmed deaths from any cause.

### 2.3 Assessment of NPAR

Venous blood samples were collected from all participants. After standardized processing, samples were frozen at −20°C and transported to certified laboratories for analysis. Neutrophil percentages were obtained from the complete blood count (CBC) profile, while serum albumin levels were measured as part of the standard biochemical assessment. NPAR was calculated using the following formula: NPAR = (neutrophil percentage (%) × 100)/albumin concentration (g/dL) (Liu and Chien, 2023).

### 2.4 Covariates

This study included various covariates related to sociodemographic characteristics, lifestyle behaviors, and health status. Demographic characteristics (sex, age, race), socioeconomic factors [marital status, education, poverty-to-income ratio (PIR)], and health-related behaviors/conditions [smoking, alcohol use, medicine use, hypertension, diabetes, cardiovascular disease (CVD)] were collected through NHANES in-home questionnaires. Racial/ethnic categorization included non-Hispanic White, non-Hispanic Black, and other race groups, while marital status was categorized as married or unmarried and others. Educational attainment was categorized into three groups: below high school diploma, high school graduate/GED equivalent, and post-secondary education. PIR was stratified into < 1.5, 1.5–3.5, and > 3.5. Medicine use refers specifically to the use of aspirin and statin medications. Smoking, alcohol use, hypertension, and diabetes were defined according to NHANES questionnaire items (SMQ020, ALQ101, BPQ020, DIQ010). CVD history was based on self-reported diagnoses of congestive heart failure (MCQ160B), coronary heart disease (MCQ160C), angina (MCQ160D), or myocardial infarction (MCQ160E), confirmed by healthcare professionals. Body mass index (BMI) was measured at mobile examination centers and classified as normal (<25.0 kg/m^2^), overweight (25.0–30.0 kg/m^2^), or obese (> 30.0 kg/m^2^). Additionally, serum alanine aminotransferase (ALT) and serum creatinine (SCr) levels were analyzed as part of laboratory assessments.

### 2.5 Statistical analysis

Demographic profiles were examined according to mortality outcomes (all-cause and cardiovascular). Statistical analyses incorporated NHANES complex survey design elements including examination weights (WTMEC2YR), stratification (SDMVSTRA), and cluster variables (SDMVPSU) to maintain national representativeness. Continuous data are reported as means with standard deviations, while categorical data are shown as counts with proportions. Weighted rank-sum and chi-square tests were employed for continuous and categorical variable comparisons, respectively.

We employed Cox regression to examine NPAR’s association with mortality outcomes in older stroke survivors, presenting findings as hazard ratios (HR) with 95% confidence intervals (CI). NPAR was evaluated as both a continuous measure and in quartile categories (Q1-Q4, reference = Q1). Multivariable analyses included: Model 1 (crude); Model 2 (sex/age/race-adjusted); Model 3 (fully adjusted for sociodemographic, lifestyle, and clinical factors).

To explore the dose-response association, including both linear and non-linear relationships, we conducted a restricted cubic spline (RCS) analysis with the 5th, 50th, and 95th percentiles of NPAR as knot points, adjusting for confounders in Model 3. Survival curves were generated using the Kaplan-Meier method to estimate survival probabilities among NPAR quartile groups, with statistical significance assessed by log-rank testing. Furthermore, time-dependent receiver operating characteristic (ROC) analyses were performed to evaluate NPAR’s prognostic capacity across different timeframes, with dynamic area under the curve (AUC) curves constructed to visualize predictive performance over time. To contextualize the prognostic performance of NPAR, we further compared it with established inflammatory biomarkers, including the neutrophil-to-lymphocyte ratio (NLR; neutrophil count/lymphocyte count), platelet-to-lymphocyte ratio (PLR; platelet count/lymphocyte count), and systemic immune-inflammation index (SII; [platelet count × neutrophil count]/lymphocyte count).

After adjusting for all covariates in the primary analytical model (Model 3), we performed stratified analyses by sex (male/female), BMI (< 25, 25–30, > 30), smoking status (yes/no), alcohol use (yes/no), diabetes (yes/no), hypertension (yes/no), and CVD (yes/no). Interaction tests were incorporated to assess potential effect modification in the association.

All statistical analyses were conducted in R Studio, adopting a two-tailed significance threshold of *P* < 0.05.

## 3 Results

### 3.1 Baseline characteristics of elderly stroke survivors

This study included 1,026 participants, weighted to represent approximately 2,909,810 elderly stroke survivors in the U.S. community. Table 1 summarizes baseline characteristics stratified by all-cause and cardiovascular mortality. The mean participant age was 72.6 ± 6.99 years, with 45.0% male and 78.2% non-Hispanic White. During a mean follow-up period of 6.65 years, 543 individuals died from all causes, and 172 from cardiovascular events. The mean NPAR level was 14.9 ± 2.74. Both mortality groups were characterized by older age, non-Hispanic White ethnicity, absence of alcohol consumption, a history of CVD, and no use of aspirin or statin medications. Furthermore, individuals with elevated all-cause mortality risk tended to exhibit reduced educational levels, decreased family earnings, lower BMI, and increased SCr and NPAR levels.

**TABLE 1:** Participant baseline characteristics stratified by all-cause and cardiovascular mortality.

Characteristics	Overall *N* = 2,909,810 *n* = 1,716	All-cause mortality		*P*-value	Cardiovascular mortality		*P*-value
		**Yes (*n* = 543)**	**No (*n* = 483)**		**Yes (*n* = 172)**	**No (*n* = 854)**	
**Age (years)**	72.6 ± 6.99	75.1 ± 6.62	70.1 ± 6.46	<0.001	74.9 ± 7.20	72.2 ± 6.87	<0.001
**Gender (%)**				0.294			0.182
Male	45.0%	47.3%	42.6%		51.2%	43.8%	
Female	55.0%	52.7%	57.4%		48.8%	56.2%	
**Race (%)**				0.001			0.034
Non-Hispanic White	78.2%	84.3%	71.9%		85.8%	76.7%	
Non-Hispanic Black	10.1%	7.6%	12.7%		6.7%	10.7%	
Others	11.7%	8.1%	15.4%		7.4%	12.6%	
**Marital status (%)**				0.053			0.604
Married	54.7%	50.6%	58.9%		52.7%	55.1%	
Unmarried and others	45.3%	49.4%	41.1%		47.3%	44.9%	
**Education level (%)**				<0.001			0.146
Less than high school	28.7%	35.5%	21.8%		31.4%	28.2%	
High school or GED	29.3%	28.7%	30.0%		21.5%	30.8%	
Above high school	42.0%	35.9%	48.3%		47.0%	41.0%	
**PIR (%)**				0.005			0.155
< 1.5	33.8%	36.0%	31.4%		38.8%	32.8%	
1.5–3.5	42.5%	46.1%	38.8%		45.0%	42.0%	
> 3.5	23.7%	17.9%	29.7%		16.2%	25.1%	
**BMI (kg/m^2^, %)**				0.018			0.489
< 25	25.3%	27.1%	23.4%		27.3%	24.9%	
25–30	34.6%	38.1%	31.1%		37.8%	34.0%	
> 30	40.1%	34.8%	45.5%		34.9%	41.1%	
**Smoking (%)**				0.482			0.717
Yes	57.8%	59.2%	56.3%		56.3%	58.0%	
No	42.2%	40.8%	43.7%		43.7%	42.0%	
**Alcohol use (%)**				<0.001			0.029
Yes	65.0%	56.9%	73.3%		55.4%	66.8%	
No	35.0%	43.1%	26.7%		44.6%	33.2%	
**Diabetes (%)**				0.182			0.027
Yes	34.1%	31.4%	36.9%		25.4%	35.7%	
No	65.9%	68.6%	63.1%		74.6%	64.3%	
**Hypertension (%)**				0.589			0.940
Yes	78.6%	77.8%	79.5%		78.9%	78.6%	
No	21.4%	22.2%	20.5%		21.1%	21.4%	
**CVD (%)**				0.001			<0.001
Yes	40.3%	46.7%	33.8%		52.3%	38.1%	
No	59.7%	53.3%	66.2%		47.7%	61.9%	
**Medicine use (%)**				<0.001			0.024
Yes	55.2%	45.8%	64.8%		44.7%	57.2%	
No	44.8%	54.2%	35.2%		55.3%	42.8%	
**ALT (U/L)**	21.6 ± 13.09	20.5 ± 10.10	22.6 ± 15.51	0.082	20.8 ± 11.95	21.7 ± 13.29	0.464
**SCr (μmol/L)**	100.0 ± 51.72	108.6 ± 63.46	91.3 ± 33.77	<0.001	105.4 ± 55.10	99.0 ± 51.03	0.145
**NPAR**	14.9 ± 2.74	15.2 ± 3.00	14.5 ± 2.40	0.004	15.3 ± 2.71	14.8 ± 2.74	0.145

Continuous variables were showed as mean ± SD, categorical variables were showed as percentage. PIR, poverty index ratio; BMI, body mass index; CVD, cardiovascular disease; ALT, serum alanine aminotransferase; SCr, serum creatinine.

### 3.2 Relationship of NAPR to prognosis in elderly stroke survivors

The Cox proportional hazards models in Table 2 demonstrate NPAR’s association with mortality outcomes in older stroke patients. Elevated NPAR levels showed statistically significant positive relationships with both all-cause and cardiovascular mortality. For all-cause mortality, NPAR as a continuous variable was significantly associated with increased risk in Model 1 (HR = 1.12, 95% CI: 1.08–1.15) and Model 2 (HR = 1.11, 95% CI: 1.07–1.14), with the association persisting in the fully adjusted Model 3 (HR = 1.09, 95% CI: 1.05–1.12). When stratified into quartiles, participants in Q4 exhibited a 69% higher risk of all-cause mortality compared to Q1 (HR = 1.69, 95% CI: 1.33–2.16, *P* for trend < 0.001). A similar pattern was observed for cardiovascular mortality, where NPAR remained significantly associated across all models (Model 1: HR = 1.13, 95% CI: 1.07–1.20; Model 2: HR = 1.12, 95% CI: 1.06–1.19; Model 3: HR = 1.11, 95% CI: 1.04–1.17). Notably, participants in Q4 had an 87% higher risk of cardiovascular mortality compared to Q1 (HR = 1.87, 95% CI: 1.19–2.93, *P* for trend < 0.001).

**TABLE 2:** Association between NPAR and mortality.

Characteristic	Model 1			Model 2			Model 3		
	**HR**	**95% CI**	***P*-value**	**HR**	**95% CI**	***P*-value**	**HR**	**95% CI**	***P*-value**
**All-cause mortality**									
**NPAR (continuous)**	1.12	1.08, 1.15	<0.001	1.11	1.07, 1.14	<0.001	1.09	1.05, 1.12	<0.001
**NPAR**									
Q1	Ref	Ref		Ref	Ref		Ref	Ref	
Q2	1.09	0.85, 1.40	0.492	0.99	0.77, 1.27	0.952	0.99	0.77, 1.28	0.934
Q3	1.31	1.02, 1.68	0.032	1.18	0.92, 1.51	0.199	1.09	0.85, 1.41	0.503
Q4	1.95	1.54, 2.48	<0.001	1.72	1.35, 2.18	<0.001	1.69	1.33, 2.16	<0.001
P for trend			<0.001			<0.001			<0.001
**Cardiovascular mortality**									
**NPAR (continuous)**	1.13	1.07, 1.20	<0.001	1.12	1.06, 1.19	<0.001	1.11	1.04, 1.17	<0.001
**NPAR**									
Q1	Ref	Ref		Ref	Ref		Ref	Ref	
Q2	1.08	0.68, 1.72	0.742	0.97	0.61, 1.55	0.904	0.96	0.60, 1.55	0.870
Q3	1.76	1.14, 2.71	0.011	1.57	1.01, 2.43	0.045	1.50	0.95, 2.34	0.079
Q4	2.21	1.43, 3.43	<0.001	1.91	1.23, 2.98	0.004	1.87	1.19, 2.93	0.006
P for trend			<0.001			<0.001			<0.001

Model 1: Unadjusted. Model 2: Adjusted for Gender, Age, and Race. Model 3: Adjusted for Gender, Age, Race, Marital status, Education level, PIR, BMI, Smoking, Alcohol use, Diabetes, Hypertension, Medicine use, CVD, ALT, and SCr.

**FIGURE 2:**
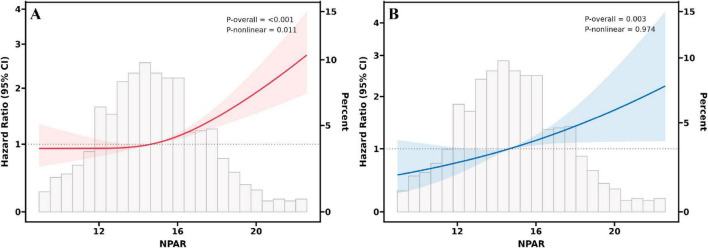
RCS fitting for the association between NPAR and all-cause **(A)** and cardiovascular **(B)** mortality.

RCS analysis provides further insights into the association between NPAR and all-cause mortality (Figure 2A) and cardiovascular mortality (Figure 2B). The solid line represents the estimated HR, with the shaded region indicating the 95% CI. The analysis revealed a non-linear, inverse L-shaped association was observed between NPAR and all-cause mortality (*P*-non-linear < 0.05), characterized by relatively stable risk at lower NPAR levels, followed by a sharp increase once the threshold is exceeded. In contrast, a linear relationship was observed with cardiovascular mortality (P-non-linear > 0.05). As shown in Table 3, threshold effect analysis identified 14.5 as a key inflection point (*P* for log likelihood ratio < 0.05). When NPAR was below 14.5, no significant association was detected, whereas NPAR values of 14.5 or higher were significantly associated with increased all-cause mortality risk (HR = 1.14, 95% CI: 1.09–1.19, *P* < 0.001).

**TABLE 3:** Threshold effect analysis of NPAR on all-cause mortality.

Characteristic	HR	95% CI	*P*-value
**Break-point = 14.5**			
NPAR < 14.5	1.00	0.93, 1.07	0.995
NPAR ≥ 14.5	1.14	1.09, 1.19	<0.001
Log likelihood ratio			0.010

Adjusted for Gender, Age, Race, Marital status, Education level, PIR, BMI, Smoking, Alcohol use, Diabetes, Hypertension, Medicine use, CVD, ALT, and SCr.

**FIGURE 3:**
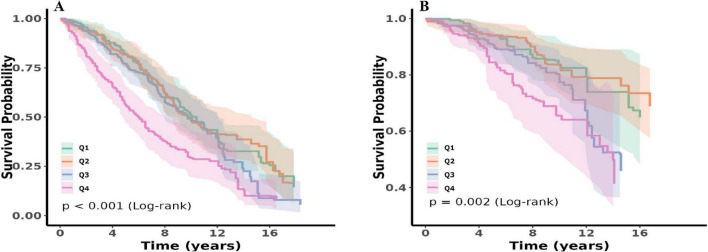
Kaplan–Meier survival analysis plot for all-cause **(A)** and cardiovascular **(B)** mortality with quartile groups of NPAR.

Kaplan–Meier survival analysis demonstrated a significant difference between NPAR quartiles and both all-cause (Figure 3A) and cardiovascular mortality (Figure 3B). Participants in Q4 exhibited significantly lower long-term survival rates than those in Q1–Q3 (*P* < 0.05, log-rank).

**FIGURE 4:**
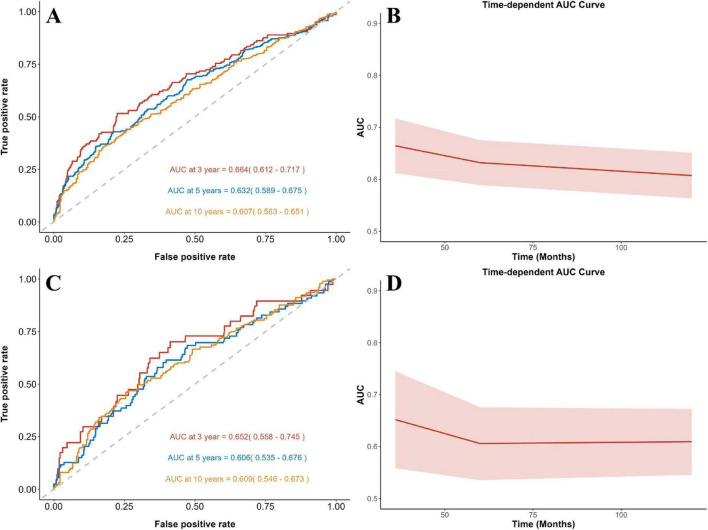
Time-dependent ROC curves and time-dependent AUC values of NPAR for predicting all-cause **(A,B)** and cardiovascular **(C,D)** mortality.

Time-dependent ROC curve analysis demonstrated that NPAR maintained a stable predictive performance for mortality (Figures 4A–D), with AUC of 0.664/0.632/0.607 (all-cause) and 0.652/0.606/0.609 (cardiovascular) at 3/5/10 years respectively. Compared to NLR, PLR, and SII, NPAR demonstrated higher predictive accuracy for both all-cause and cardiovascular mortality. Notably, NLR showed a higher AUC for 10-year cardiovascular mortality. However, NPAR consistently outperformed all other biomarkers across all remaining endpoints (Supplementary Figures 1-3).

**FIGURE 5:**
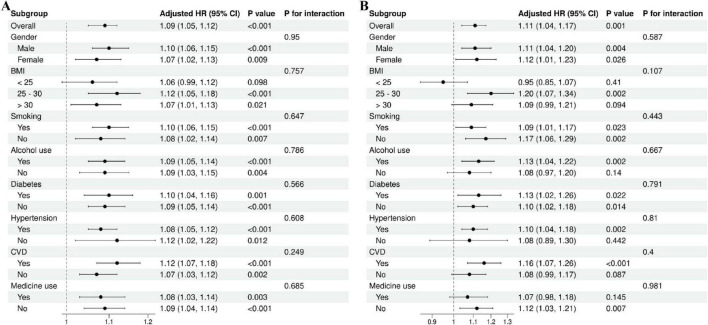
Subgroup analysis of NPAR with all-cause **(A)** and cardiovascular **(B)** mortality.

In addition, we employed forest plots to illustrate the findings from subgroup analyses and interaction tests (Figures 5A,B). The findings revealed no significant heterogeneity among subgroups (all *P* for interaction > 0.05), further affirming the robustness and consistency of these associations across diverse populations.

## 4 Discussion

In this cohort of 1,026 individuals, elevated NPAR levels showed significant mortality risk associations. Restricted cubic spline modeling identified an inverse L-shaped association with all-cause mortality, with an inflection point at NPAR = 14.5. Survival curves demonstrated markedly reduced survival probabilities for participants in the top NPAR quartile. Time-dependent receiver operating characteristic analyses confirmed NPAR’s strong predictive capacity for mortality outcomes across 3-, 5-, and 10-year intervals. Subgroup analyses confirmed the consistency of this association across diverse population subgroups. To the best of our knowledge, this is the first study to demonstrate a significant association between NPAR and mortality among community-dwelling older stroke survivors in the United States.

Recent research has highlighted a strong association between NPAR and stroke risk. A large-scale cross-sectional study of 56,566 NHANES participants identified a non-linear positive correlation between NPAR and stroke risk (OR = 1.09, 95% CI: 1.05–1.12) (Xu et al., 2024). Similarly, Ye et al.’s analysis of 48,734 participants showed 62% greater stroke risk in the highest versus lowest NPAR quartile (OR = 1.62, 95% CI: 1.40–1.89), with this association remaining consistent across subgroups stratified by age and comorbidities, reinforcing the robustness of NPAR as a cross-population risk marker (Ye et al., 2025). Furthermore, Yang et al.’s retrospective analysis revealed that higher NPAR levels independently predicted 3-month stroke recurrence (OR = 9.71, 95% CI 3.05–31.62) in first-time acute ischemic stroke patients and, importantly, outperformed both neutrophil-to-lymphocyte ratio (NLR) and standard inflammatory/nutritional markers in predictive accuracy, highlighting its clinical utility for early risk stratification (Yang et al., 2024). Collectively, these findings establish NPAR as a key predictor of stroke onset and recurrence. However, the clinical significance of this inflammation-nutrition composite biomarker likely extends beyond risk stratification. In elderly stroke survivors, chronic inflammation and nutritional imbalance may accelerate atherosclerosis, promote multi-organ dysfunction, and ultimately contribute to increased mortality.

The association of NPAR with mortality has been extensively validated across various populations. A cohort study of 817 ICU patients with iron-deficiency anemia demonstrated an inverse L-shaped non-linear relationship between NPAR and both 30-day and 1-year mortality, with this trend remaining highly consistent across different age groups and comorbidity subgroups (Lin et al., 2025). Similarly, Tan et al.’s analysis of 2,699 NHANES participants with diabetic nephropathy confirmed that higher NPAR levels were strongly linked to elevated mortality (HR = 2.17, 95% CI 1.83-2.58) (Tan et al., 2025). These results align with our findings, reinforcing the robust link between NPAR and mortality risk. However, notable differences emerge when comparing our study with the retrospective analysis by Chen et al. on ICU stroke patients (Chen et al., 2022). While both studies confirmed the relationship between elevated NPAR and increased mortality risk, our study identified a significantly lower threshold (14.5 vs. 25.0) and a distinct association pattern (inverse L-shaped non-linear vs. fixed-group linear). These discrepancies may be attributed to differences in study populations (community-dwelling elderly vs. critically ill ICU patients) and the varying severity of inflammation-nutrition imbalance. ICU patients typically exhibit severe systemic inflammation and hypoalbuminemia, which may elevate the NPAR threshold, whereas in community-dwelling elderly individuals, chronic low-grade inflammation may trigger mortality risk at lower NPAR levels. Importantly, the NPAR threshold of 14.5 identified in our study may serve as a clinically useful reference point for early risk stratification. Individuals with NPAR values exceeding this cutoff may be considered at higher mortality risk and could benefit from enhanced clinical attention. Specifically, this threshold may help guide decisions regarding the need for more proactive nutritional support or anti-inflammatory strategies in older stroke survivors. In addition to its independent prognostic value, NPAR may complement existing clinical scoring systems used for stroke prognosis, such as the National Institutes of Health Stroke Scale (NIHSS) and the Essen Stroke Risk Score (ESRS). While these scores primarily assess neurological function or vascular comorbidity burden and recurrence risk, NPAR reflects systemic inflammation and nutritional status, offering a distinct but clinically relevant dimension. Integrating NPAR into multivariable models alongside established clinical scores could enhance overall prognostic accuracy and improve individualized risk stratification in older stroke survivors. Additionally, our findings are consistent with previous studies highlighting the superior prognostic value of NPAR compared to traditional inflammatory index (Liu et al., 2024; Zhu et al., 2025). This may stem from NPAR’s unique integration of both inflammatory and nutritional pathways, whereas NLR, PLR, and SII solely reflect immune cell dynamics.

While previous studies have firmly established the association between NPAR and mortality risk, the underlying pathophysiological mechanisms require further investigation. Neutrophils, as key effectors of innate immunity, play a pivotal role in post-stroke inflammation by releasing reactive oxygen species (ROS), matrix metalloproteinase-9 (MMP-9), and neutrophil extracellular traps (NETs), thereby exacerbating blood-brain barrier disruption, cerebral edema, and atherosclerotic plaque instability (Tang et al., 2024). Notably, excessive NET formation has been directly linked to larger infarct size and worse neurological outcomes (Li et al., 2022). In parallel, declining serum albumin levels not only reflect nutritional depletion but also weaken antioxidant defenses and endothelial integrity (Soeters et al., 2019; Belinskaia et al., 2021). As a composite biomarker of inflammatory burden and nutritional status, elevated NPAR signifies a state of “high inflammation and low defense,” which synergistically promotes endothelial dysfunction, immune-metabolic dysregulation, and multi-organ failure, ultimately heightening mortality risk (Guo et al., 2024). These mechanistic insights underscore the potential of targeting NPAR through anti-inflammatory therapies and nutritional interventions as a strategic approach to improving the prognosis of elderly stroke survivors.

Our research exhibits multiple advantages. First, being the inaugural population-based longitudinal investigation examining NPAR’s relationship with mortality outcomes in community-dwelling geriatric stroke survivors. Second, the use of Cox regression models with extensive covariate adjustments minimizes confounding bias, ensuring the robustness of our findings. Moreover, subgroup analyses stratified by sex, BMI, and comorbidities demonstrated consistent associations between NPAR and mortality risk across diverse populations, underscoring its potential as a broadly applicable biomarker, particularly in resource-constrained healthcare environments. Finally, as NPAR is derived from routine blood tests, it offers a cost-effective and highly accessible prognostic tool for primary care settings. Notwithstanding these merits, certain study limitations warrant consideration. As NHANES does not provide information on the timing of medication intake relative to blood sampling, we were unable to determine whether inflammatory markers were measured before or after the administration of agents such as aspirin or anticoagulants. This limitation may introduce variability in NPAR levels due to the acute effects of these medications on neutrophil counts or albumin concentrations. This may introduce potential variability in NPAR levels due to the acute effects of medications on neutrophil count or albumin levels. Furthermore, stroke history relied on self-report without subtype classification (ischemic vs. hemorrhagic), which may reduce clinical interpretability given differing pathophysiological profiles and mortality risks between subtypes. Lastly, the single-timepoint measurement of NPAR inherently limits our ability to capture dynamic fluctuations in inflammatory-nutritional status. Serial NPAR assessments could better reflect disease progression trajectories and identify critical windows for intervention. Future studies incorporating longitudinal NPAR monitoring are needed to establish its temporal relationship with outcomes and optimize risk prediction.

## 5 Conclusion

In summary, this study is the first to demonstrate, within a community cohort of elderly stroke survivors, that higher NPAR values independently predict greater mortality. These findings underscore the potential of NPAR as a critical biomarker for risk stratification and targeted interventions in elderly stroke survivors. Future multicenter prospective studies are warranted to explore combined anti-inflammatory and nutritional intervention strategies based on dynamic NPAR monitoring, thereby optimizing community-based post-stroke management.

## Data Availability

The datasets presented in this study can be found in online repositories. The names of the repository/repositories and accession number(s) can be found at: www.cdc.gov/nchs/NHANEs/.
