# Supplementary material for: Association of neutrophil-percentage-to-albumin ratio with mortality in older stroke survivors
Source: Front Aging Neurosci. 2025 May 30;17:1611289. doi: 10.3389/fnagi.2025.1611289 (PMC12162721; doi:10.3389/fnagi.2025.1611289)

Fig S1. Time-dependent ROC curves and time-dependent AUC values of NLR for predicting all-cause (A-B) and cardiovascular (C-D) mortality.


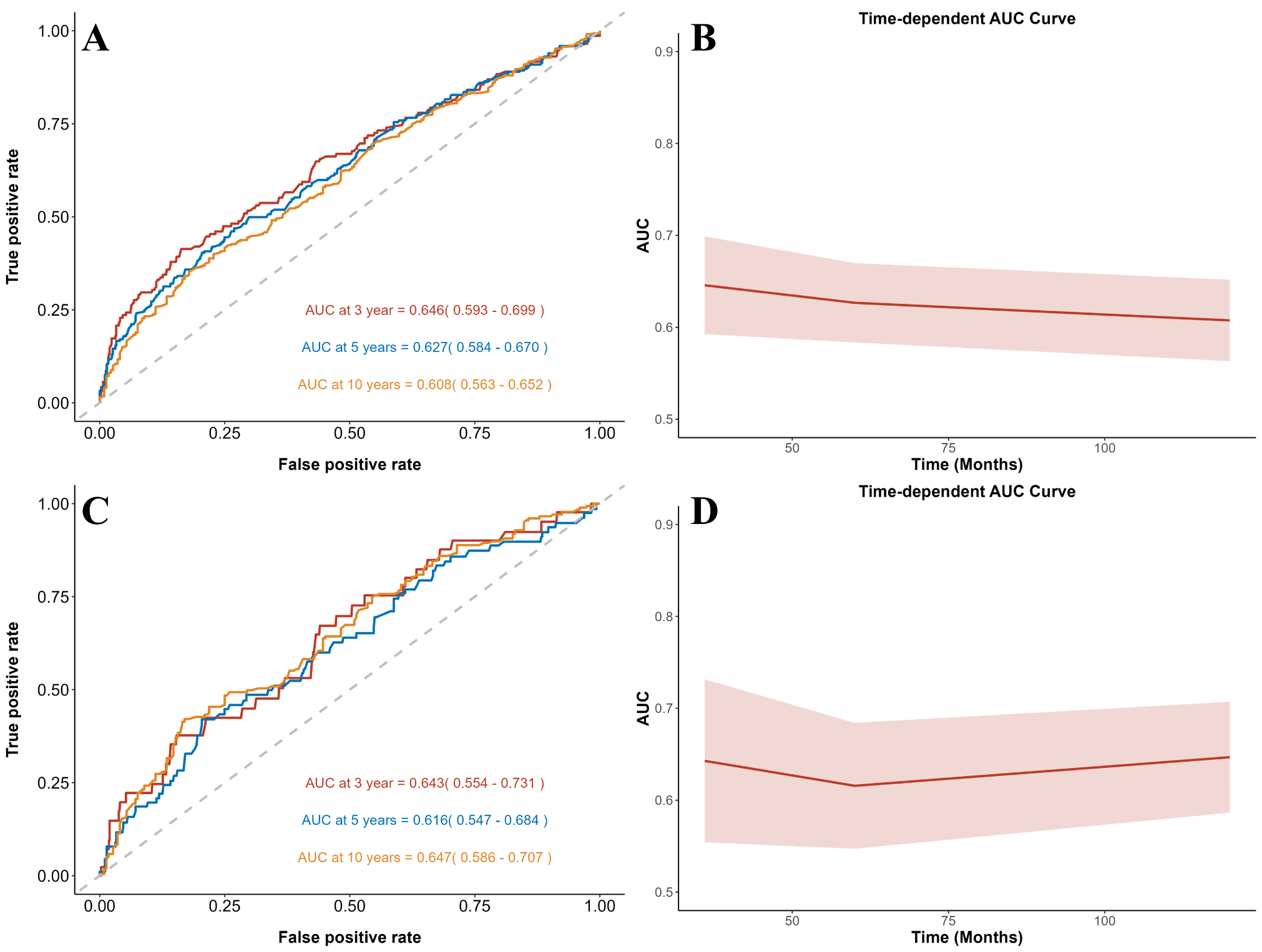


Fig S2. Time-dependent ROC curves and time-dependent AUC values of PLR for predicting all-cause (A-B) and cardiovascular (C-D) mortality.


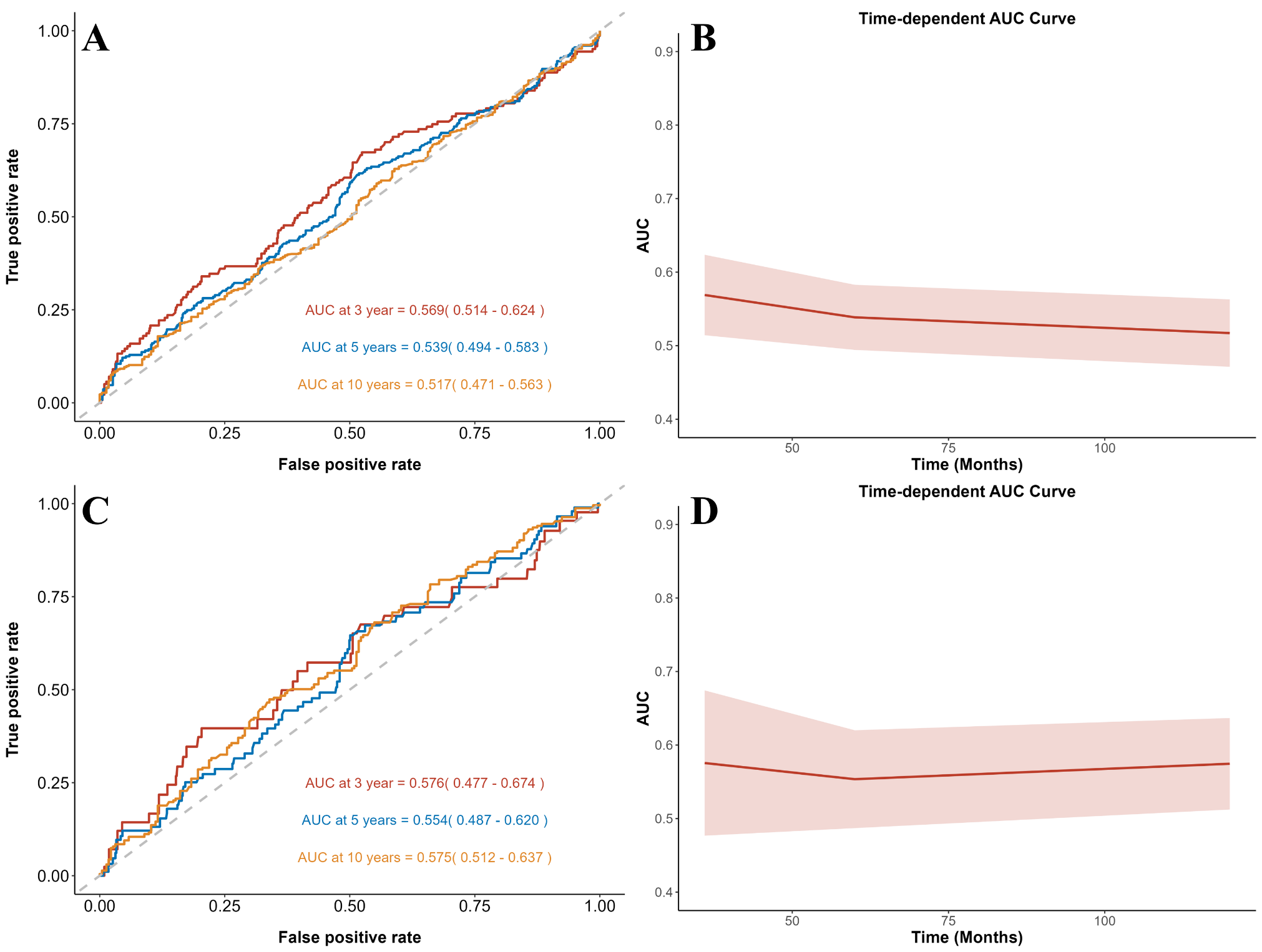


Fig S3. Time-dependent ROC curves and time-dependent AUC values of SII for predicting all-cause (A-B) and cardiovascular (C-D) mortality.


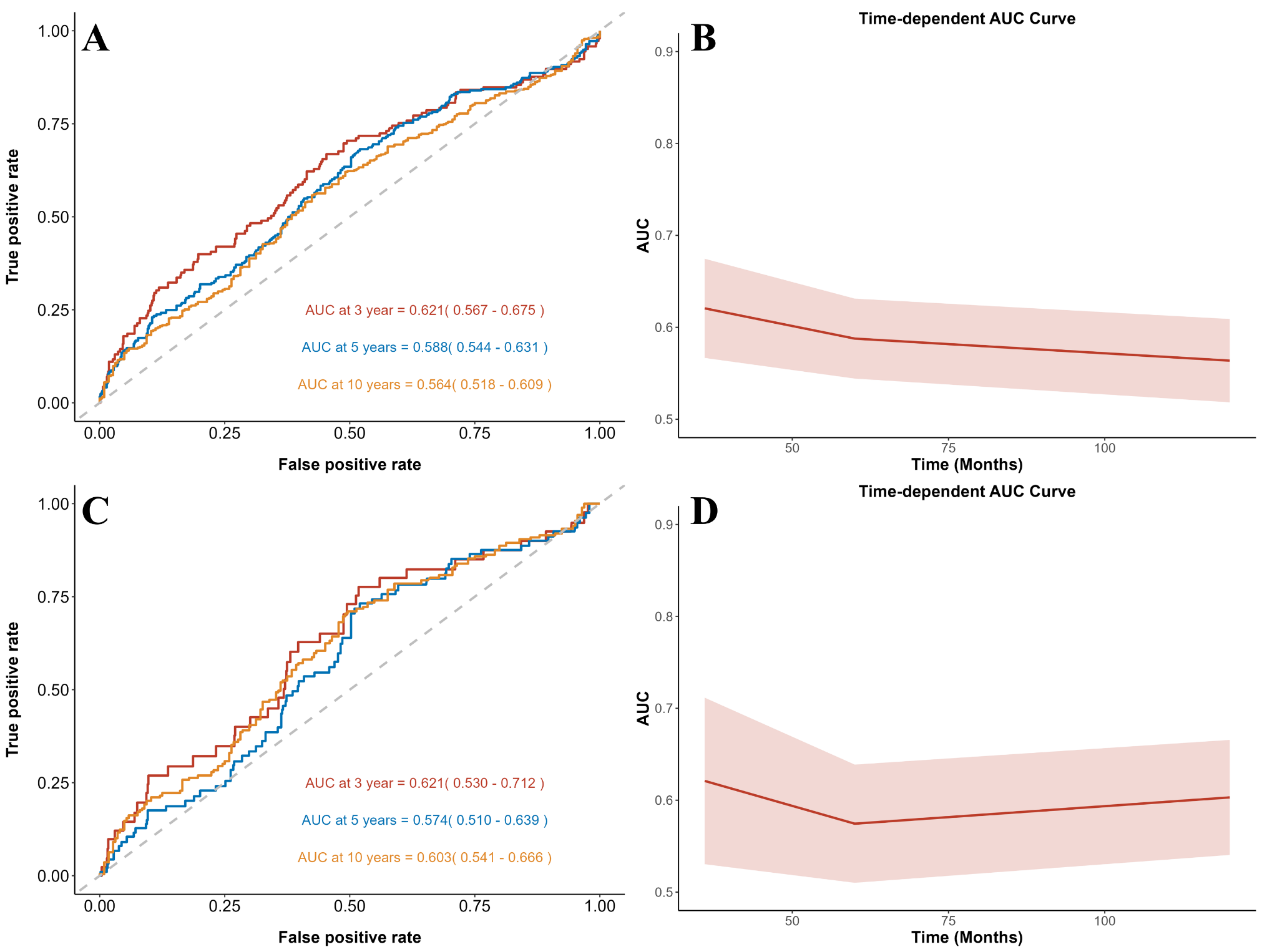

Supplement: Supplementary file 1 [file Data_Sheet_1.docx]
